# Supplementary material for: Endothelial-to-Mesenchymal Transition in Atherosclerosis: Friend or Foe?
Source: Cells. 2022 Sep 21;11(19):2946. doi: 10.3390/cells11192946 (PMC9563961; doi:10.3390/cells11192946)
Supplement: Supplementary file 1 [file cells-11-02946-s001.zip › cells-1899803-supplementary.pdf]

Endothelial-to-Mesenchymal Transition in Atherosclerosis: Friend or Foe?

Sarin Gole[1], Svyatoslav Tkachenko[2], Tarek Masannat[1], Richard A. Baylis[3], and Olga A. Cherepanova[1,*]

[1] Cardiovascular and Metabolic Sciences, Lerner Research Institute, Cleveland Clinic, 9500 Euclid Avenue, NB5, Cleveland, 44195, USA
[2] Genetics and Genome Sciences, Case Western Reserve University, 2109 Adelbert, RD, BRB, Cleveland, 44106, USA
[3] Department of Medicine, Massachusetts General Hospital, 55 Fruit St Gray 730, Boston, MA, 02114, USA
* Correspondence: cherepol@ccf.org; Tel.: +1-216-445-7491

Supplementary Data

Cell Ranger Processing of Human Data from Wirka et al. [30].
Publicly available single-cell RNA sequencing data for four patient samples (proxi-mal-to-mid right coronary artery: 65-year-old male; 54-year-old male; 65-year-old male, and 58-year-old female) have been obtained from the Gene Expression Omnibus data-base with the SuperSeries reference number GSE131780. Sra files were obtained using the prefetch command from sratoolkit (version 2.10.8), and fastq files were extracted us-ing the fastq-dump command (from the same toolkit) with split-files option. Count matrices were extracted from the fastq files, and initial clustering was obtained using the "cellranger count" command (cellranger version 3.0.0) with the human genome, assembly GRCh38. The initial clustering for integrated samples from the cellranger is shown on panel (a) of Figure 2, overlaid with the expression of CDH5 and PECAM1 genes that were used as markers of EC cells. Based on these markers, three clusters were deemed to be carrying the majority of EC cells. Before further analysis, quality control (QC) was performed, and cells were filtered based on three criteria: library size, number of detected genes, and percentage of reads belonging to mitochondria (QC was performed separately for biological/technical replicates [see Table below for details]). After that, the filtered data were run through an R script, based on the Seurat R package. Next, samples were run through the Seurat pipeline normalizing and integrating the data. Then cells corresponding to three "EC clusters" from the cellranger clustering (see above) were extracted from the integrated object, clustered anew, and subjected to the UMAP dimensionality reduction (Figure 2b). Expression levels of ACTA2 and FN1 across the new Seurat-generated clusters were obtained and plotted in Figure 2c and Figure 2d, respectively.

Table – QC parameters:

GSM	Library size*	Genes**	Mitochondria reads***
gsm3819856	1000	300-2500	9%
gsm3819857	800	275-2500	10%
gsm3819858	700	300-2600	12%
gsm3819859	800	300-2500	9%
gsm3819860	850	300-3000	7%
gsm3819861	950	300-3000	6%
gsm3819862	1000	350-2800	6%
gsm3819863	950	300-3000	7%

* Cells with library size above this value are accepted
** Cells with the number of detected genes between these values are accepted
*** Cells with mitochondria read percentage below this are accepted
